# Plasmacytoid Urothelial Carcinoma of the Urinary Bladder Metastatic to the Duodenum: A Case Report—Diagnostic Relevance of GATA3 Immunohistochemistry

**DOI:** 10.1155/2017/5209059

**Published:** 2017-01-31

**Authors:** Hermann Brustmann

**Affiliations:** Department of Pathology, Landesklinikum Baden-Moedling, Baden, Austria

## Abstract

Plasmacytoid urothelial carcinoma (PUC) of the urinary bladder is a rare and aggressive subtype of urothelial carcinoma. Its deceptive morphology is characterized by a discohesive growth of cells with plasmacytoid morphology. Since this tumor might be confused with plasmacytoma, lymphoma, or carcinoma variants, appropriate diagnosis in small biopsy samples could be challenging. This study reports the case of a 53-year-old man who presented with frequent nocturnal urgency, without hematuria. A transurethral bladder and a prostate resection specimen displayed infiltration of neoplastic cells in a spray-like discohesive pattern with occasional formation of small irregular nests and cord-like arrangements. The basic morphology of the tumor cells was plasmacytoid, with eccentric nuclei and eosinophilic cytoplasm. Tumor cells grew through the lamina muscularis mucosae, with splintering of the bladder wall musculature and infiltration of prostatic tissue. They displayed strong and diffuse nuclear reactivity for p53 and GATA3. Eight months after surgery, the patient experienced upper abdominal discomfort. A duodenal biopsy showed infiltration of plasmacytoid atypical cells strongly immunoreactive for GATA3, consistent with the previously diagnosed PUC. The patient died eleven months after the primary diagnosis of his PUC of tumor cachexia losing about 50% of his original body weight, furthermore, with ascites and intraperitoneal tumor spread.

## 1. Introduction

Plasmacytoid urothelial cancer (PUC) is a rare and aggressive variant of urothelial carcinoma (UC) which was only adopted by the World Health Organization (WHO) classification in 2004 [1, 2]. Less than 100 cases have been reported in the literature; thus, these tumors are poorly characterized [3]. PUC is notable for a discohesive growth of cells with plasmacytoid morphology displaying eccentric nuclei and eosinophilic cytoplasm, frequently extending in the bladder wall and in the perivesical adipose tissue [4]. As a result, the outcome appears to be poorer compared to conventional high-grade UC [1, 5]. A predilection of PUC for intraperitoneal noncontiguous spread has been reported [6]. Since this tumor may be confused with plasmacytoma, lymphoma, or carcinoma variants, appropriate diagnosis in small biopsy samples may be challenging [7].

## 2. Case Report

A 53-year-old man presented at a local urologist with frequent nocturnal urgency. His medical history was unremarkable for relevant diseases. He indicated occasional cigarette smoking. There were no signs of hematuria. Ultrasound examination of the urinary tract revealed a thickened wall of the urinary bladder. This finding was verified by computed tomography. A transurethral resection of the presumed suspicious lesion was performed; at the same time prostatic tissue was resected too, for a preliminary diagnosis of benign prostatic hyperplasia. These specimens were submitted to this author's institution for histological examination.

**Figure 1:**
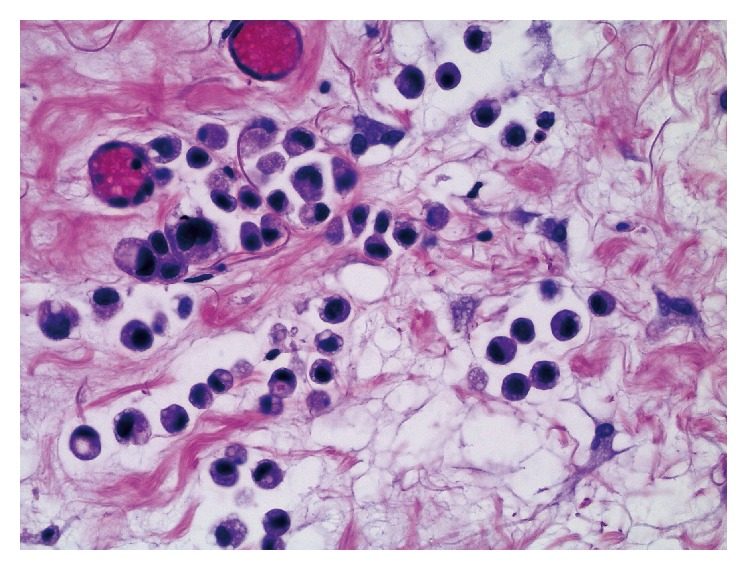
Transurethral bladder resection specimen displaying plasmacytoid tumor cells, with eccentric nuclei and eosinophilic cytoplasm. There is occasional intracytoplasmic vacuolization (H&E, ×400).

**Figure 2:**
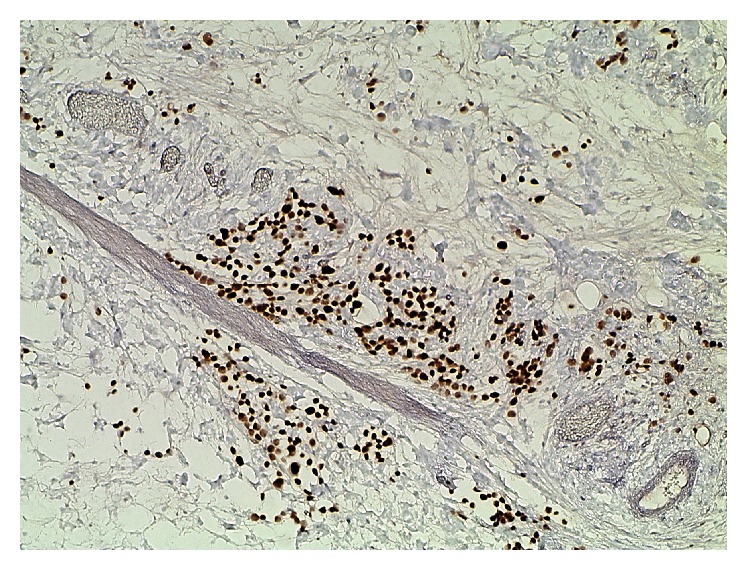
Strong and diffuse nuclear immunoreactivity for GATA3 by tumor cells in the transurethral bladder resection specimen (×400).

The transurethral bladder resection specimen consisted of gray-brown tissue fragments of 3.7 cm in diameter and the prostate resection specimen of chip-like tissue pieces of 2.5 cm in diameter. Both specimens displayed infiltration of neoplastic cells in a spray-like discohesive pattern with occasional formation of small irregular nests and cord-like arrangements. There were erosive defects of the bladder urothelium; the suburothelial stroma was widened with tumor cells growing through the lamina muscularis mucosae and splintering of the bladder wall musculature. On H&E staining, the basic morphology of the tumor cells was plasmacytoid, with eccentric nuclei and eosinophilic cytoplasm (Figure 1). Occasional tumor cells were multinuclear in eccentric fashion, with a strongly eosinophilic broad cytoplasm, consistent with rhabdoid morphology. There was intracytoplasmic vacuolization sometimes. Invasion of lymphatic vessels was observed. Superficial small areas of typical urothelial in situ cancer were identified. These tumor formations were also noted in the second specimen, with infiltration of prostatic tissue. These features were suspicious for a high-grade subtype of urothelial carcinoma, and thus immunohistochemistry was performed with a Ventana Benchmark Ultra using antibodies to bcl-2 (mouse monoclonal, ready-to-use, Ventana), keratin 20 (rabbit monoclonal, ready-to-use, Ventana), desmin (mouse monoclonal, ready-to-use, Ventana), p16 (mouse monoclonal, ready-to-use, Ventana), p40 (mouse monoclonal, ready-to-use, Ventana), GATA3 (mouse monoclonal, ready-to-use, Cell Marque), p53 (mouse monoclonal, ready-to-use, Ventana), and OSCAR pankeratin (mouse monoclonal, ready-to-use, Cell Marque). Tumor cells displayed both strong and diffuse nuclear reactivity for p53 and GATA3 (Figure 2). There was diffuse and strong reactivity for OSCAR pankeratin, occasional and weak nuclear p40 staining, partial keratin 20 staining, and bcl-2 reactivity in about 10% of tumor cells. Morphology and immunohistochemical staining patterns were consistent with a final diagnosis of a poorly differentiated urothelial carcinoma with plasmacytoid and occasional rhabdoid features.

**Figure 3:**
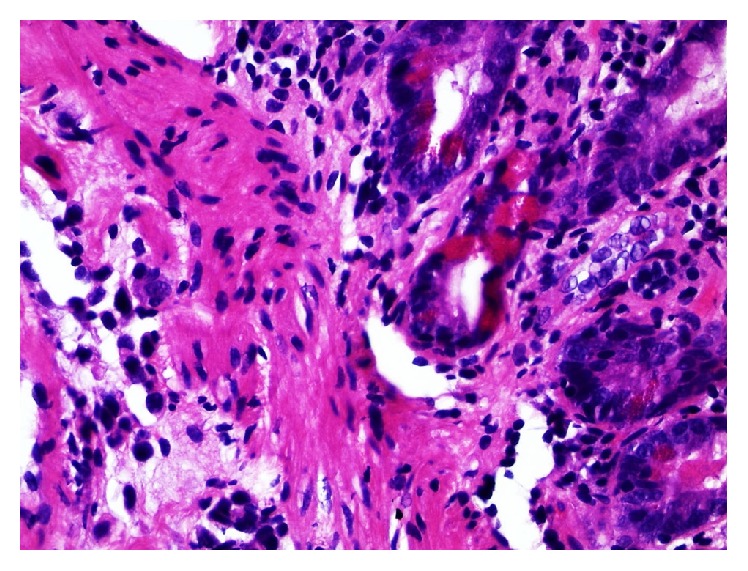
Duodenal biopsy with atypical cells in submucosal location, showing enlarged nuclei and nucleoli, with partial plasmacytoid morphology. These cells were presented in a single cell pattern (H&E, ×400).

**Figure 4:**
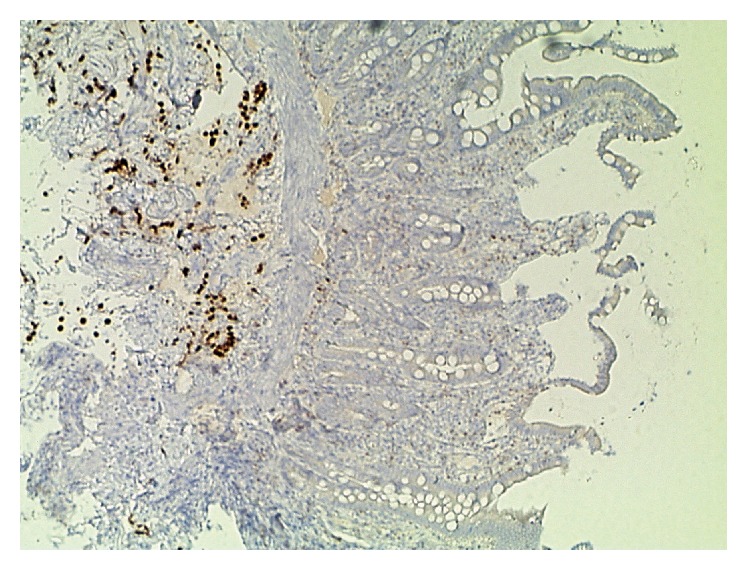
Duodenal biopsy showing strong nuclear GATA3 immunoreactivity in many tumor cells, which are observed more readily on immunostaining than on H&E histology (×100).

The patient received surgery at another institution, with cystoprostatectomy, lymphadenectomy, and an ileal conduit. Histological examination confirmed the above described diagnosis. There were isolated tumor cells noted in lymph nodes as well as at the resection margin. Additionally, an acinar adenocarcinoma of the prostate (pT2c, Gleason 3 + 3) was reported. That institution decided against further therapy. The patient went to a rehabilitation clinic and did well; he gained weight again and was in a positive psychologic state. About eight months after surgery, the patient complained about upper abdominal discomfort and pain. He vomited dark red-brown to greenish materials. Gastroesophageal biopsies showed erosive inflammation of the gastroesophageal junction. However, standard treatment for this disease did not improve the patient's condition. A computed tomography was performed. There were no pathological findings in the thoracic organs. Some acidic fluid was noted in the abdominal cavity. The intrahepatic biliary tract showed ductal ectasias in the left lobe. However, there was no evidence of a mass in the pancreas or biliary tract. After a tumor board discussion with the pathologist (H.B.), who emphasized the spray-like growth pattern of the known PUC, a decision for an endoscopic retrograde cholangiopancreatography (ERCP) was made. During ERCP, the papilla of Vater was not traceable. The duodenum was distorted, hardly passable, and the mucosa was livid bluish. Four biopsy pieces measuring 5 mm in diameter together were received for histological examination. Histologically, the slides showed duodenal mucosa with a normal villous architecture and a mildly active inflammation. Only one biopsy piece displayed a narrow rim of adjacent submucosa. Some sparse interspersed atypical cells were noted in this layer, showing enlarged nuclei and nucleoli, with partial plasmacytoid morphology (Figure 3). These cells were presented in a single cell pattern. They were immunoreactive for OSCAR pankeratin and GATA3 and, thus, were considered consistent with the previously diagnosed PUC (Figure 4). The patient's condition deteriorated rapidly and a tumor board recommendation was delivered after discussion with the patient and his family for best supportive care. The patient died eleven months after the primary diagnosis of his PUC of tumor cachexia, losing about 50% of his original body weight, with ascites and intraperitoneal tumor spread. No autopsy was performed. The patient's widow agreed to the presentation of her husband's disease in a case report.

## 3. Discussion

Due to its plasmacytoid morphology, PUC may pose difficulties in differential diagnostic considerations. It is not surprising that such lesions were previously considered plasmacytomas or lymphomas [3]. Moreover, these plasmacytoid tumor cells were reported positive for CD138, which is considered an immunohistochemical marker for plasma cells [4, 8]. The differential diagnosis includes other cancers with discohesive growth patterns like diffuse gastric carcinoma or lobular breast cancer [3]. In those cancers intracytoplasmic vacuolization may be present in PUC [7]. Since the case presented herein was both associated with urothelial carcinoma in situ, as well as there being an awareness of the entity of PUC, a CD138 immunostaining was not done. However, the nature of this tumor was studied und confirmed by immunohistochemical markers. A pankeratin staining done in our case by OSCAR antibody has been noted positive in 97% of PUC cases previously [1]. Recently, GATA3 (endothelial transcription factor 3) has been shown of value in UC including PUC [4]. GATA3 was diffusely positive in the tumor cells of our case. Histological and immunohistological findings thus confirmed the diagnosis of PUC. p40, an isoform of p63 and reactive in many UC, was of no particular diagnostic use.

In this case, GATA3 immunostaining was of extreme importance at the final presentation of this patient with intraabdominal metastases. The diffuse growth pattern of rather sparse tumor cells in the duodenal submucosa was closely mimicking a diffuse type of gastrointestinal carcinoma. However, clinical history and judicious choice of immunohistochemical antibodies in a limited biopsy sample helped to both arrive at the correct diagnosis and to prevent unnecessary surgical treatment for an upper abdominal cancer. Zhao et al. reported on a high specificity of GATA3 as a diagnostic marker in UC including PUC, with not only maintained, but increased expression in regional metastases [9]. Liang et al. studied the differential expression of GATA3 in UC variants and described it as a useful marker for confirming the urothelial origin of micropapillary and plasmacytoid variants of UC but not that of sarcomatoid or small cell variants [10]. Others observed GATA3 expression in 88% of UC variants including micropapillary, plasmacytoid, nested, clear cell, and microcystic tumors [11]. Thus, GATA3 appears to be an appropriate marker in the differential diagnosis [11] and useful in the recognition of the urothelial lineage of PUC in metastatic settings, as described in the case at hand. This view is supported by recent studies. Miettinen et al. [12] published a study on GATA3 expression in epithelial and nonepithelial tumors. They reported that GATA3 was expressed in >90% of primary and metastatic ductal and lobular breast, urothelial, and cutaneous basal cell carcinomas, as well as trophoblastic and endodermal sinus tumors. In another study, cholangiocarcinomas and gastric carcinomas only weakly and sporadically expressed GATA3 [13].

PUC is notable for a predilection for intraperitoneal spread. One study reported that 33% of PUC presented with intraperitoneal disease and 20% had subsequent metastases involving serosal surfaces and, furthermore, emphasized the possibility of noncontiguous intraperitoneal spread involving serosal surfaces, which is an important feature in order to ensure proper staging and clinical follow-up [6]. The architectural patterns of PUC may vary. The cells may present in cords and single cells, small nests, solid sheet-like and diffuse discohesive patternless arrangements [7]. These aggressive growth patterns indicate the associated poor prognosis of PUC [7]. Dayyani et al. found that the most common site of PUC recurrence was the peritoneum and that in some cases an initial surge in the serum CA-125 levels preceded radiologic and symptomatic findings of progression. Thus, they described the peritoneum as the primary site of recurrence and suggested a follow-up with serial CA-125 measurements [5]. However, they concluded that the prognosis of PUC remains poor, with few long-term survivors despite neoadjuvant chemotherapy. Additionally, they emphasized the high risk of relapse in the peritoneal lining and suggested that peritoneal carcinomatosis should be considered in patients presenting with abdominal symptoms [5]. Our case was discussed by clinicians as a possible intrahepatic bile duct carcinoma in the differential diagnosis at presentation with upper abdominal symptoms. Noting the biological behavior of the primary PUC by the pathologist and subsequent duodenal biopsy leads to the correct recognition of the tumor recurrence. Rice et al. suggested that the PUC variant of UC may be a marker for locally advanced and aggressive disease rather than specifically influencing lymphatic spread, with a high incidence of positive surgical margins [14].

In conclusion, to the best of the author's knowledge, this case is the first PUC reported with duodenal metastases diagnosed on a duodenal biopsy specimen. One previous study described a PUC metastatic to the stomach and duodenum as suspected on CAT scan [3]. In that case, a gastric biopsy was thought to be consistent with poorly differentiated gastric carcinoma initially, and gastrectomy was done. There is no note of a duodenal biopsy in that manuscript [3]. Our case ran a rapidly fatal course. Clinical history is extremely helpful in the correct interpretation of an unusual biopsy site like the duodenum. GATA3 immunohistochemistry is a valuable tool in the differential diagnostic evaluation of a PUC, be it primary or metastatic, and should be included in a panel of antibodies in such cases.
